# Systematic review with network meta-analysis of randomized controlled trials of robotic-assisted arm training for improving activities of daily living and upper limb function after stroke

**DOI:** 10.1186/s12984-020-00715-0

**Published:** 2020-06-30

**Authors:** Jan Mehrholz, Alex Pollock, Marcus Pohl, Joachim Kugler, Bernhard Elsner

**Affiliations:** 1 Department of Public Health, Dresden Medical School, Technical University Dresden, Dresden, Germany; 2 Nursing Midwifery and Allied Health Professions (NMAHP) Research Unit, Glasgow Caledonian University, Glasgow, UK; 3 Vamed Klinik Schloss Pulsnitz, Pulsnitz, Germany

**Keywords:** Stroke, Rehabilitation, Robots, Upper limb, Network meta-analysis

## Abstract

**Background:**

The aim of the present study was to to assess the relative effectiveness of the various types of electromechanical-assisted arm devices and approaches after stroke.

**Method:**

This is a systematic review of randomized controlled trials with network meta-analysis. Our primary endpoints were activities of daily living (measured e.g. with Barthel-Index) and hand-arm function (measured e.g. with the Fugl-Meyer Scale for the upper limb), our secondary endpoints were hand-arm strength (measured e.g. with the Motricity Index) and safety. We used conventional arm training as our reference category and compared it with different intervention categories of electromechanical-assisted arm training depending on the therapy approach. We did indirect comparisons between the type of robotic device. We considered the heterogeneity of the studies by means of confidence and prediction intervals.

**Results:**

Fifty five randomized controlled trials, including 2654 patients with stroke, met our inclusion criteria.

For the primary endpoints activities of daily living and hand-arm function and the secondary endpoint hand-arm strength, none of the interventions achieved statistically significant improvements, taking into account the heterogeneity of the studies.

Safety did not differ with regard to the individual interventions of arm rehabilitation after stroke.

**Conclusion:**

The outcomes of robotic-assisted arm training were comparable with conventional therapy.

Indirect comparisons suggest that no one type of robotic device is any better or worse than any other device, providing no clear evidence to support the selection of specific types of robotic device to promote hand-arm recovery.

**Trial registration:**

PROSPERO 2017 CRD42017075411

## Introduction

Stroke is one of the most common diseases worldwide and leads to permanent disability, reduced quality of life and thus to a high burden of disease [1]. A majority of stroke patients have limited hand and arm function and are therefore restricted in their daily activities [2]. The recovery of hand-arm function is therefore an important goal for rehabilitation after stroke [1]. In recent years, interventions such as electromechanical-assisted arm training have been introduced to improve hand and arm functions [3, 4]. It has been argued that use of electromechanical-assisted arm training can support the provision of evidence-based rehabilitation, by facilitating therapy that is intensive, frequent and repetitive [3]. However, while systematic reviews show some beneficial effects of electromechanical-assisted arm training on upper limb motor function, these effects are not clinically relevant [3, 4]. Furthermore, there is also some evidence of a detrimental effect, with one systematic review concluding that muscle tone of the upper limb might be negatively influenced by robotic-assisted arm-training [4].

The devices used in electromechanically-assisted arm therapy target the motor function of either the shoulder/elbow, elbow/wrist, wrist/hand, hand/finger or the entire upper extremity [3, 5]. There are two broad types of electromechanical devices which have been used to enable or assist arm and/or hand movement in a patient with a paretic limb following stroke:

- An external robotic arm, known as an exoskeleton, which is designed to control one or more joints of the paretic arm. The exoskeleton uses torque actuators in order to apply rotational forces to move, or assist the movement, at a joint. For example, a robotic arm could support the weight of a patient’s arm in the horizontal plane, and assist combined movement at the shoulder and elbow [5].
- A robotic device, known as an end-effector, which assists movement of only the distal part of the paretic arm [3, 4]. These devices generally only have contact with the patient’s hand/fingers; and move – or assist the movement – of the distal part of the arm, which may result in movement at more proximal parts of the arm. End effectors may act to move just the paretic limb, or may act to support bilateral arm movement. For example, an end effector may comprise two handles, which are held by the patient’s hands. Movement of the handles facilitates bilateral pronation/supination of the forearm and flexion/extension of the wrist. Movement of the patient’s affected arm may be passive, either driven entirely by the robot or by active movement of the unaffected arm, or may be active-assisted, supported by the robot or unaffected arm [5].

In addition to generating either passive or assisted movement of a paretic arm, electromechanically assisted arm therapy can give patients feedback about the joint position and the arm power used.

Electromechanical-assisted arm therapy may, alternatively, be classified based on whether the robot acts: more proximally or distally, with a one-sided / unilateral or double-sided / bilateral exercise approach, or to give support to specific joint sections. End effector-based therapy robots generally initiate movement via contact with the patient’s hand, generating movement of more proximal joints from this distal contact; while exoskeletal devices can directly guide and control movement of both proximal and distal joints via series of drive elements.

Furthermore, the torque actuators which can be used within robotic devices may have different mechanisms of action, and there is ongoing debate regarding these different approaches to control of force. For example, it remains unclear whether a compliant actuator (e.g. series elastic actuators, an elastic element attached) is any more beneficial than an assist-as-needed control mechanism (e.g. which encourages patients’ active participation), or an impedance control mechanism (e.g. an end effector that takes into account the kinematics and dynamics of the object being manipulated).

With a rapid growth in new technologies and devices over recent decades, there are now a large number of different electromechanical-assisted arm training devices designed to move, or assist movement of, the arm. The types of therapy provided by different devices differ significantly both in terms of the technologies employed and the therapy provided. There is a growing body of evidence, synthesized within systematic reviews, that demonstrates that electromechanical-assisted arm training may be beneficial for recovery of arm function after stroke, with quality of the evidence judged to be ‘high’ (using the GRADE approach) [3, 4]. However, although the evidence on robotic-assisted arm training after stroke seems robust, there remains a lack of information about the relative effects of different types of devices. The existing systematic reviews are arguably limited by their narrow focus, for example on the effectiveness of robotic-assisted arm training or electromechanical-assisted arm rehabilitation compared to control interventions [3, 4, 6]. Thus, while in practice it is crucial to know which type of robotic device performs most effectively in a given situation, the current evidence base lacks direct comparisons of two or more different types of device. Furthermore, it remains unclear which of the different devices or approaches may be most effective for certain subgroups of patients with stroke, meaning that a treating clinician will encounter difficulties in deciding which specific form of treatment to select and/or apply for a specific patient after stroke. Thus, while systematic reviews have explored the effectiveness of electromechanical-assisted arm rehabilitation [3, 4], these have not directly compared the effects of the different types of devices or therapy provided by devices, in order to determine the optimal type of electromechanical-assisted arm training for individual patients.

An approach to solving this problem is offered by network meta-analyses. These enable quantitative synopsis of an “evidence network” by combining direct and indirect effects of three or more interventions, compared to the same comparative intervention (often a control or a no-treatment intervention), within a randomized controlled trial [7]. This is also called a multiple treatment comparison [8].

In this way, network meta-analyses allow the quantitative synthesis of evidence of effectiveness of interventions directly compared within the same randomised controlled trial (direct comparisons) and interventions from different randomised controlled trials which have a common comparator (indirect comparisons) [7]. Network meta-analyses could therefore provide an efficient method for determining the relative effects of different electromechanical-assisted arm training devices and therapy approaches, without the need for new randomised controlled trials.

The aim of the present study was therefore to provide a systematic overview of current randomised controlled trials of electromechanical-assisted arm training, and to use network meta-analysis to assess the relative effectiveness of the various types of electromechanical-assisted arm devices and approaches. We aimed to evaluate the relative effect of different types of electromechanical-assisted arm training on activities of daily living, hand/arm function and hand/arm strength in patients with stroke, and to explore the safety of these devices.

## Methods

### Study protocol and registration

We registered a study protocol for the present study, which is published in the PROSPERO database under ID CRD42017075411 according to PRISMA criteria [9].

### Role of the funding source

There was no funding source for this study.

### Eligibility criteria

We included published and unpublished studies with adults after stroke (by clinical definition). We included all randomized controlled trials with parallel group design and randomized cross-over studies comparing any type of electromechanical-assisted arm training with any other intervention. For inclusion, studies had to measure at least one of our pre-stated outcomes of interest at the end of the intervention period. Our primary outcomes of interest were measure of activities of daily living (ADL) and hand/arm function. Our secondary outcomes of interest were hand/arm strength and safety after stroke.

For our primary outcome of ADL we accepted measurements taken using the Barthel Index [10] or the Functional Independence Measure (FIM) if they were available. These outcome measurement scales were prioritised as we anticipated that these would be reported by the majority of our included studies. If these scales were not available, we accepted other scales that measured ADL. Although our primary outcome of interest was ADL, it was not necessary for a study to either state that they aimed to improve ADL, or to include a measure of ADL, in order to be included.

For our primary outcome of hand-arm function we prioritised reports of the Fugl-Meyer-Scale for the upper limb [11]. We chose to focus on the Fugl-Meyer scale because there is evidence that 2/3 of studies in this field report this [3]. If this scale was not available, we accepted other scales that measured hand-arm function such as the Wolf Motor Function Test or shoulder-disability scales; but where the Fugl-Meyer scale was available we did not extract data from any other hand/arm function scales. A short overview of the included studies, and the assessment used for hand-arm-function is shown in Table 1 (Table 1: Overview of categories of included studies and devices).

**Table 1:** Overview of categories of included studies and devices

Study ID	Intervention	device	Assessment Hand-Arm Function
Abdullah 2011	UPAHT	industrial robot	CAHAI-7
Ang 2014	DGFHT	Haptic knob	UE-FM
Brokaw 2014	EXAHT	ARMin III, HandSOME	UE-FM
Burgar 2011	UPAHT	MIME	UE-FM
Bustamante 2016	UDFHT	Robot Gym (TheraDrive)	UE-FM
Conroy 2011	UPAHT	InMotion 2.0	UE-FM
Daly 2005	UPAHT	InMotion	UE-FM
Fazekas 2007	EPAHT	REHABROB	UE-FM
Grigoras 2016	DGFHT	Hybrid FES Exoskelett	UE-FM
Hesse 2005	EBAHT	Bi-Manu Track	UE-FM
Hesse 2014	EBAHT	Bi-Manu Track	UE-FM
Hollenstein 2011	EXAHT	Armeo	UE-FM
Housman 2009	EXAHT	T-WREX	UE-FM
Hsieh 2011	EBAHT	Bi-Manu Track	UE-FM
Hsieh 2014	EBAHT	Bi-Manu Track	UE-FM
Hwang 2012	UDFHT	Amadeo	UE-FM
KlamrothMarg anska 2014	EXAHT	ARMin	UE-FM
Kutner 2010	UDFHT	Handmentor	SIS hand function
Lee 2016	UPAHT	NEURO X System	Manual function test
Liao 2011	EBAHT	Bi-Manu Track	UE-FM
Lo 2010	UPAHT	MIT Manus	UE-FM
Lum 2006	UPAHT	MIME	UE-FM
Masiero 2007	EPAHT	NEREBOT	UE-FM
Masiero 2011	EPAHT	NEREBOT	UE-FM
Mayr 2008	EXAHT	ARMOR	CMSA
McCabe 2015	UPAHT	InMotion 2.0	UE-FM
Orihuela- Espina 2016	UDFHT	Amadeo	UE-FM
Rabadi 2008	UPAHT	MIT-Manus MITManus/InMotion 2.0	UE-FM
Sale 2014	UPAHT	Amadeo	UE-FM
Stein 2017	UDFHT	Hand-exoskelett (self made)	UE-FM
Susanto 2015	DGFHT	Reogo	UE-FM
Takahashi 2016	UPAHT	HapticMaster	UE-FM
Timmermans 2014	EPAHT	Armassist	UE-FM
Tomic 2017	UPAHT	Gloreha	UE-FM
Vanoglio 2017	DGFHT	Gloreha	Quick-DASH
Villafane 2017	DGFHT	MIT-Manus	Quick-DASH
Volpe 2000	UPAHT	In Motion 2.0	UE-FM
Volpe 2008	UPAHT	Hand Mentor per	UE-FM
Wolf 2015	UDFHT	Bi-Manu Track	UE-FM
Wu 2012	EBAHT	Reogo	UE-FM
Yoo 2013	UPAHT	RT-AAN	WMFT
Cho 2019	UPAHT	NMES ROBOT	UE_FM
Qian 2017	EPAHT	Inmotion	UE_FM
Hung 2019	UPAHT	Bi-Manu Track	UE_FM
Hung 2019	EBAHT	Armeo Spring	UE_FM
Daun 2018	EXAHT	RA- Shoulder	UE_FM
Kim 2019	EPAHT	therapy	K_SDQ
Lee 2018	EPAHT	REJOYCE Rob	UE_FM
RATULS 2019	UPAHT	MIT-Manus	UE_FM
Conroy2019	UPAHT	Inmotion	UE_FM

For our secondary outcome of hand-arm strength we accepted the scales such as the Motricity Index [12], grip strength and equivalent scales and versions. However, if these scales were not available, we accepted other scales that measured hand-arm muscle strength. Where more than one measure of hand/arm strength was reported, we used a pre-specified list to inform selection; this list first prioritized the Motricity Index and second prioritized grip strength, as we anticipated that the majority of studies would have used these scales.

### Information sources

We searched the Cochrane Stroke Group’s Trials Register (last searched July 2019), the Cochrane Central Register of Controlled Trials (CENTRAL) (the Cochrane Library 2019, Issue 6), MEDLINE (1950 to July 2019), Embase (1980 to July 2019), CINAHL (1982 to July 2019), AMED (1985 to July 2019), SPORTDiscus (1949 to July 2019), PEDro (searched July 2019), Compendex (1972 to July 2019), and Inspec (1969 to July 2019). We also handsearched relevant conference proceedings, searched trials and research registers (clinicaltrials.gov last searched July 2019), checked reference lists, and contacted trialists, experts, and researchers in our field, as well as manufacturers of commercial devices for unpublished studies.

### Search

The search strategy for MEDLINE can be found in our Additional file 1. This search strategy was adapted for the other databases.

### Study selection

One reviewer (BE) considered titles and excluded obviously irrelevant studies. Two independent reviewers (BE and JM) applied selection criteria to the abstracts and full texts of remaining studies. Differences were resolved through discussion, involving a third reviewer where necessary.

### Data collection process and data items

Two independent reviewers (BE and JM) extracted the following information from each included study:

- participants (country, number of participants, age, gender, type of stroke, time from stroke onset to entry to the study, inclusion and exclusion criteria);
- comparison (details of the intervention in treatment and control groups, details of cointervention(s) in both groups, duration of treatment);
- outcomes and time points of measures (number of participants in each group and outcome, regardless of compliance)
- methods of generating randomisation schedule;
- method of concealment of allocation;
- blinding of assessors;
- adverse events and dropouts for all reasons;

Any discrepancies were resolved through discussion.

### Categories of robotic-arm training

Prior to study onset, we defined categories of different types of robotic arm-training. Informed by an existing systematic review for robotic-arm training [3], review authors discussed different possible robotic approaches and reached consensus on intervention categories. Our rationale and aim was, from a clinical point of view, to clearly define distinguishable groups of robotic-assisted arm training interventions. Our goal was to identify less than eight distinct intervention categories which could be used within this study.

We initially reached agreement that key clinical decision making generally needed to consider the following key aspects of intervention delivery: unilateral or bilateral robotic arm training, end effector-assisted or exoskeleton assisted robotic arm training, emphasis on proximal or distal arm training, glove-finger-based or not, and the combination of these approaches.

We then pre-defined the following categories of electromechanical-assisted arm training interventions used in the studies:

- UDFHT, unilateral distally emphasized finger/hand training
- EPAHT, end effector-assisted proximal emphasised unilateral arm/hand training
- UPAHT, unilaterally proximal emphasized arm/hand training
- EXAHT, exoskeleton assisted unilateral arm/hand training
- DGFHT, unilateral distal glove -based finger/hand training
- EBAHT, end effector assisted distal and bilateral arm/hand training.

We defined conventional arm training as any other control intervention which used arm training to improve ADL, without electromechanical-assisted arm training intervention.

Two reviewers independently categorised the interventions within each included study, with any differences resolved through discussion, involving a 3rd reviewer if necessary.

A short overview of the included studies, type of the device, and intervention category is shown in Table 1 (Table 1: Overview of categories of included studies and devices).

### Geometry of network

The geometry of the network characterized the relation and accuracy of the direct comparisons. We produced network diagrams [13] in order to assess of network geometry. Each intervention was represented by a node in the network. Direct comparisons between interventions were shown by lines connecting the nodes. The thickness of the line in the network graphs represents the amount of studies included for this comparison. The different colours indicate the risk of bias among the trials for each of the three dimensions (randomization sequence, concealment of randomization sequence, and blinding) as a covariable at study level in network diagrams.

### Risk of bias within individual studies

Two authors (JM and MP) independently assessed the methodological quality of the included trials using the Cochrane ‘Risk of bias’ tool (using the categories, random sequence generation,

allocation concealment, and blinding of outcome assessor as high, low or unclear risk of bias) [14]. We provided all details about the characteristics and the methodological quality of each included study in tables (Additional file 2: characteristics of studies and Additional file 3: Risk of bias of included studies). As described above, the risk of bias of the individual studies was represented within the network diagrams by using different colours.

### Summary measures

When trials used the same test procedure (e.g., Barthel Index), we calculated mean differences (MD) and the corresponding 95% confidence intervals (CI). If various result measures were used for a given endpoint, we calculated standardized mean differences (SMD) with 95% CI. For dichotomous endpoints we determined the index of the risk difference (RD) with 95% CI.

If trials used a cross-over design we used the data from the first phase of the study (i.e. the period before cross-over).

We generated contrast-based forest plots for all comparisons. We compiled a relative ranking of the competing interventions on the basis of their surface under the cumulative ranking line (SUCRA) [15]. The SUCRA values give the percentage efficacy of each individual intervention in comparison with an “ideal” treatment. All statistical analyses were performed using the software STATA SE Version 15.0 [16, 17].

### Planned method of analysis

This network meta-analysis was conducted according to a frequentist approach with weighted least squares based on a multivariate regression with random effects. This approach enables adequate consideration of multiple-arm studies and includes restricted maximum-likelihood estimation [18].

### Assessment of inconsistency

To test for possible infringement of the transitivity assumption, we assessed global inconsistency by accommodating a consistency and an inconsistency model [18, 19]. Transitivity means there are no systematic differences among the various arms of the individual studies. At local level we used the node-splitting approach [18, 20]. Alongside the quantitative tests, we performed qualitative verification of the description of the trials included with regard to important effect modifiers.

### Risk of bias across studies

We assessed the risk of bias among the trials for each of the three dimensions (randomization sequence, concealment of randomization sequence, and blinding) as a co-variable at study level in network diagrams.

### Additional analyses

We compiled a relative ranking of the competing interventions on the basis of their surface under the cumulative ranking line (SUCRA). The SUCRA values give the percentage efficacy of each individual intervention in comparison with an “ideal” treatment.

The P-score of an intervention, which may range from 0 to 1 and, can be interpreted as the mean certainty of its superiority and describes the mean degree of certainty about a particular treatment being better than another treatment.

We viewed generation of the randomization sequence, concealment of the allocation sequence, and blinding of the investigators as potentially important methodological effect modifiers and integrated them into a sensitivity analysis (Additional file 3).

## Results

### Study selection

**Fig. 1:**
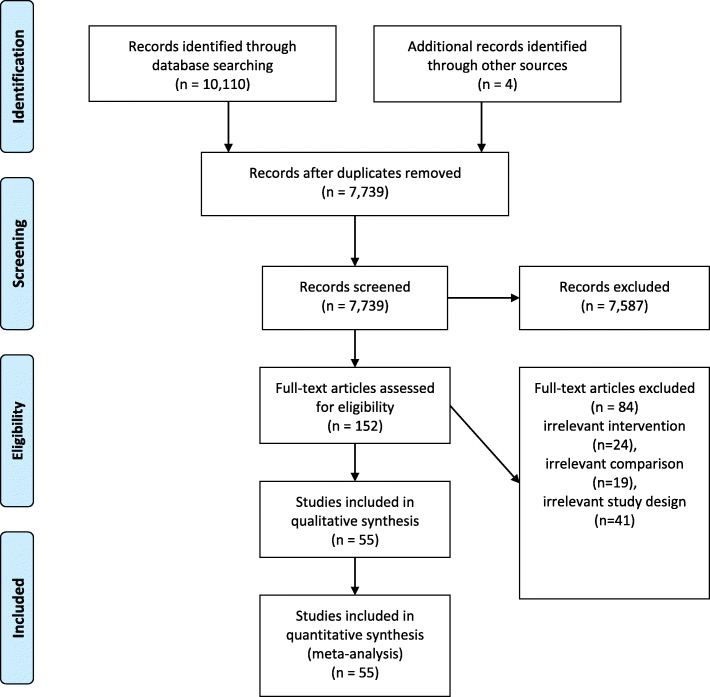
PRISMA Flow Chart

Our systematic search found 6744 matches after removing duplicates. After excluding irrelevant records 55 randomized controlled trials met our selection criteria, with a total of 2654 patients and were suitable for inclusion within our statistical meta-analyses (Fig. 1, flowchart).

### Study characteristics

Of the 55 trials meeting our inclusion criteria, 53 (96%) were randomized controlled trials and the remaining 2 trials (4%) were randomized cross-over trials. Number of participants included in the trials ranged from 8 to 770, with a mean of 24 participants. The mean age of participants ranged from 44 to 76 years, and the mean time since stroke from 14 days to 4 years.

### Presentation of network structure

**Fig. 2:**
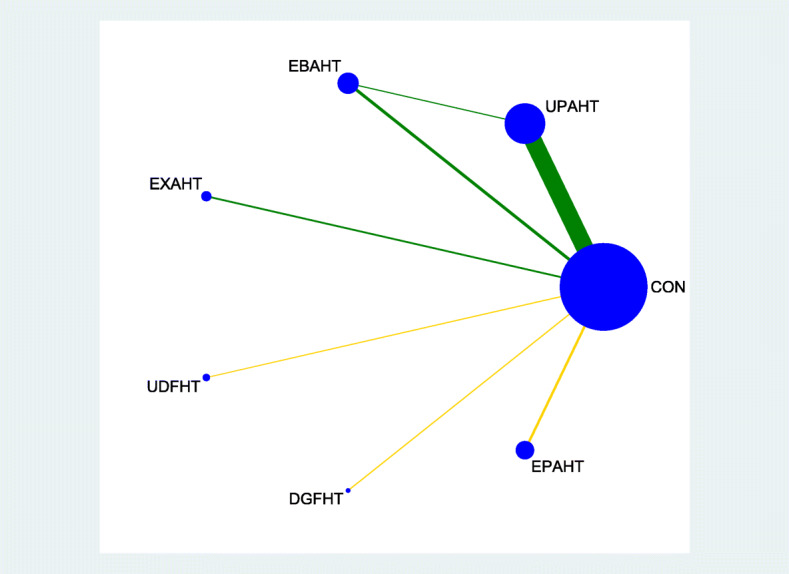
network graph_ADL

**Fig. 3:**
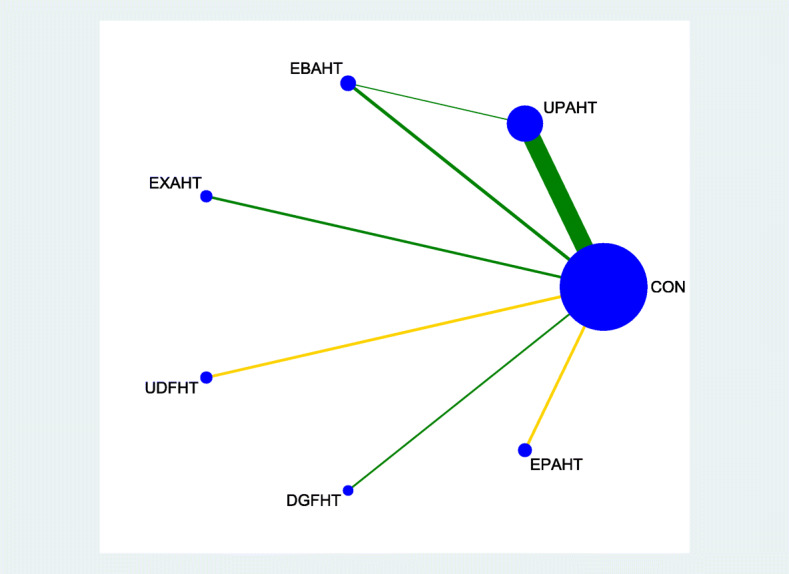
network graph_Hand_Arm_Function

**Fig. 4:**
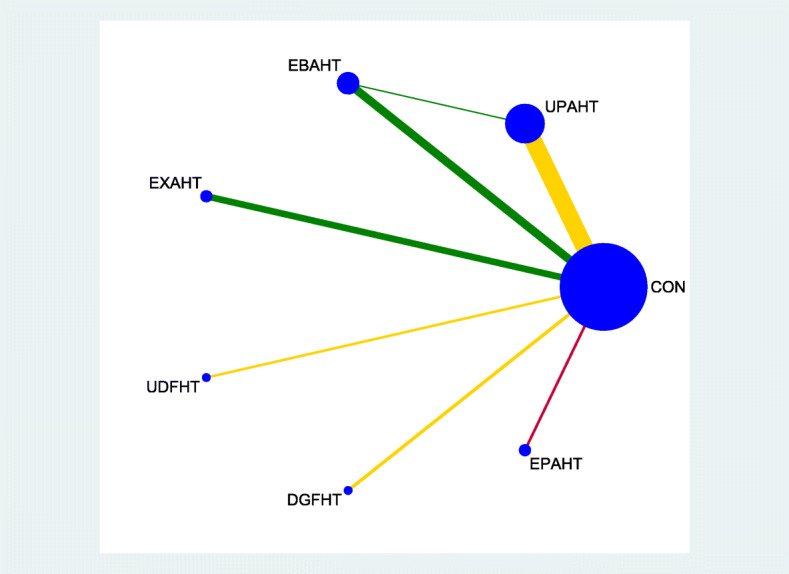
network graph_Arm strength

**Fig. 5:**
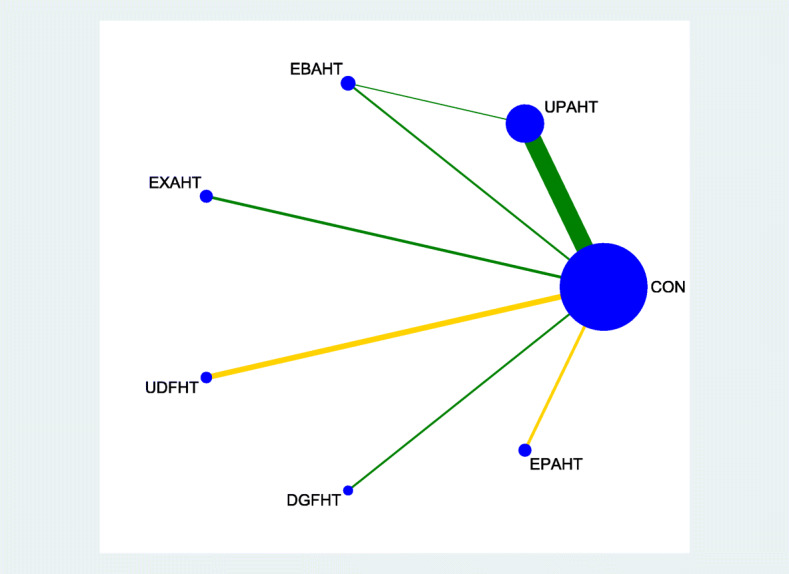
network graph_AEs

Figures 2, 3, 4 and 5 provide network graphs, illustrating the volume of evidence from RCTs comparing different types of electromechanical-assisted arm training with conventional hand-arm therapy without devices (comparator).

Figure 2 and Fig. 3 shows a network graph for the primary endpoints activities of daily living and hand-arm function, respectively. Figure 4 and Fig. 5 shows a network graph for the secondary endpoints hand-arm strength and safety.

The thickness of the lines in Figures 2, 3, 4 and 5 illustrate that the majority of direct comparisons were of UPAHT with control. The line thickness also highlights that there were more measures of muscle strength for comparisons of EBAHT and EXAHT with control than measures of ADL or hand-arm function. There was only one direct comparison of different robotic devices (EBAHT vs UPAHT); the trial which had this comparison also included a direct comparison with a control group.

### Summary of network geometry

All studies compared an active electromechanical-assisted arm training group with an active control group and a total of 28 different devices were used (as shown in Additional file 2). Most commonly used were the MIT-Manus / InMotion in ten studies (18%), the Bi-Manu-Track in seven studies (13%) and the Amadeo in three studies (5%). The active control was a form of conventional hand-arm therapy, not using any robotic devices, in all of the studies (Additional file 2). The time spent within conventional therapy was practically the same as the time spent using a robotic device.

The electromechanical-assisted arm training was categorised as UDFHT in 8 study arms; EPAHT in 8 study arms; UPAHT in 23 study arms; EXAHT in 7 study arms; DGFHT in 5 studies and EBAHT in 7 study arms (some studies used two or more treatment arms; Additional file 2).

The primary outcome of activities of daily living was measured in 30 studies, including 1857 participants receiving electromechanical-assisted arm training, and hand-arm function was measured in in 50 studies, including 2456 participants.

The secondary outcome of muscle strength of the paretic arm was measured in 24 studies, with 839 participants receiving electromechanical-assisted arm training, and 55 studies, with 2654 participants, described safety endpoint data.

### Synthesis of results

**Fig. 6:**
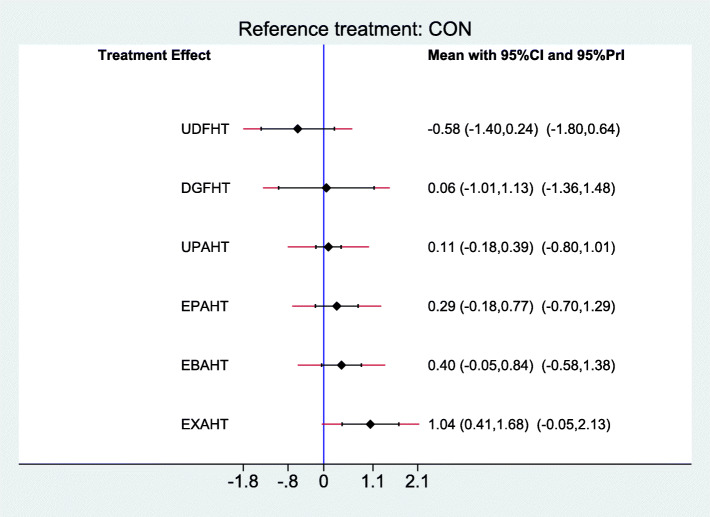
Forest Plot ADL

**Fig. 7:**
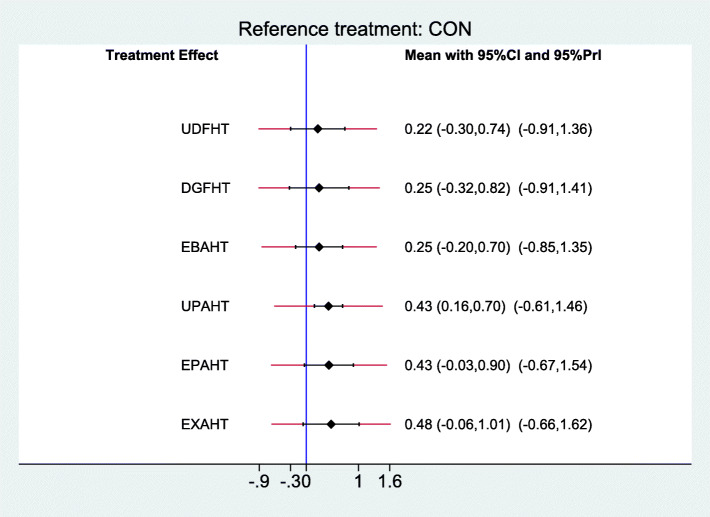
Forest Plot Hand_Arm_Function

**Fig. 8:**
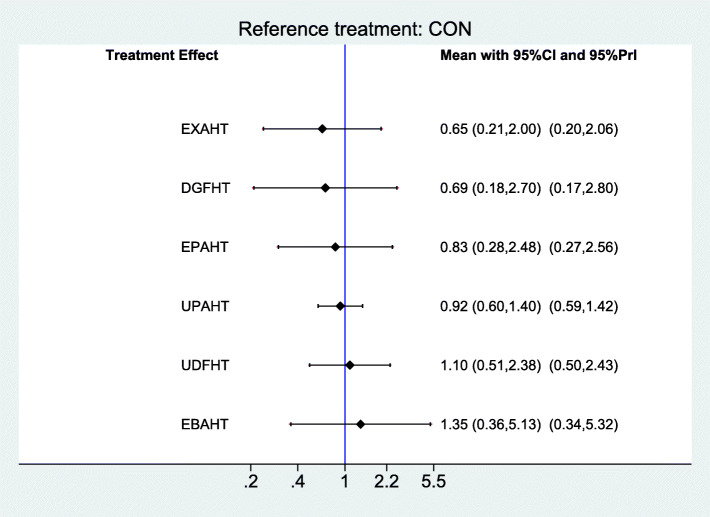
Forest Plot AEs

Results of comparisons of different categories of electromechanical-assisted arm training with conventional training without devices for outcomes of activities of daily living, hand-arm function, and safety are reported in Figs 6, 7 and 8. Figure 6 shows the forest plot of electromechanical-assisted arm training for improving ADL capacity. Figure 7 shows the forest plot of electromechanical-assisted arm training for improving hand-arm function. Figure 8 shows the forest plot of electromechanical-assisted arm training for adverse events.

For the primary outcome of activities of daily living, exoskeleton assisted unilateral arm/hand training (EXAHT) achieved significant improvements (standardized mean difference (SMD) = 1.04, 95-% confidence interval (CI): 0.41, 1.68). Taking into account the heterogeneity of the studies (95-% predictive intervals, e.g. EXAHT: − 0.05, 2.13), however, none of the intervention categories significantly improved ADL measures (Fig. 6). Indirect comparisons of the different types of electromechanical-assisted arm training did not identify any one type of intervention which was significantly more effective at improving activities of daily living than any other type of intervention (supplement).

For the primary outcomes of hand-arm functions, only unilateral proximal emphasized arm/hand training (UPAHT) achieved significant improvements (SMD = 0.43, 95%CI: 0.16, 0.70). Considering the heterogeneity of the studies (95% predictive intervals, e.g. UPAHT: − 0.61, 1.46), however, none of the interventions significantly improved hand-arm functions (Fig. 7). Indirect comparisons of the different types of electromechanical-assisted arm training did not identify any one type of intervention which was significantly more effective at improving hand-arm function than any other type of intervention (Additional file 4).

For the secondary outcome of hand-arm strength, no network-meta analysis could be done due to inconsistency in the network.

For the secondary outcome of safety, we found no systematic differences in the individual interventions (Fig. 8). Indirect comparisons of the different types of electromechanical-assisted arm training did not identify any one type of intervention which was significantly more effective at improving hand-arm strength or was significantly safer than any other type of intervention (Additional file 4).

### Exploration for inconsistency

Significant inconsistency, which means disagreement between direct and indirect comparisons, was not observed for the endpoints ADL, function and safety neither by global nor local examination (χ^2^ 0.60, *p* = 0.74; χ^2^ 0.00, *p* = 1.0 and χ^2^ 0.05, *p* = 0.97, respectively. There was excess inconsistency for the endpoint hand-arm strength (χ^2^ 6.81, *p* = 0.03), so we did not perform an analysis for this outcome.

### Risk of bias across studies

Thirty-four of the 55 included studies (62%) reported adequate generation of the random sequence, 15 of the 55 included studies (27%) reported adequate allocation sequence concealment and 41 of the 54 included studies (75%) reported adequate blinding of the outcome assessors. The risk-of-bias assessments for each study are shown in Additional file 3.

### Results of additional analyses

The results of a compiled relative ranking of the competing interventions on the basis of their surface under the cumulative ranking curve (SUCRA) can be found in Additional file 5.

We provided a table with a description of all adverse events or drop outs that occured in included studies (Additional file 6).

To investigate the effects of using different methods or tools to assess our primary outcome of hand-arm function, we performed subgroup analyses to compare different outcomes used in studies on our primary outcome hand-arm function (Additional file 7: e.g. subgroup of 41 studies with 2244 participants using the Fugl-Meyer Armtest). There was only one study with 21 participants (ID: Kutner 2010) using SIS hand function and only one study with 22 participants (ID: Yoo 2013) using the Wolf Motor Function Test. Therefore for these outcome measures no NMA was possible. There were further upper limb assessments used in studies (e.g. K-SDQ in one study with 38 participants; ID: Kim 2019, or the Quick-DASH in two 2 studies with 59 participants; ID: Vanoglio 2017 & ID: Villafane 2017; or CMSA in one study with 8 participants; ID: Mayr 2008 and other assessments) but no subgrouping was possible here.

To investigate the effects of different severities of arm paresis we compared three subgroups (Additional file 8), however, we did not find an effect of different severities of arm paresis.

To investigate the effects of different durations of illness (acut and subacute versus chronic phase after stroke) we compared two subgroups (Additional file 9). However, we did not find an effect between patients in the first 3 months or later after stroke.

## Discussion

Our systematic review with a network meta-analysis included a total of 55 studies with 2654 patients. We directly and indirectly compared the effects in a network meta-analysis comparing six categories of 28 different electro-mechanical devices for improving ADL and hand-arm function after stroke. We did not find any systematic differences in any outcomes between different approaches to hand-arm training after stroke. Overall, the number of side effects was low in all studies, indicating that safety was therefore good.

Our network meta-analyses provide new and valuable insights into the relative effects of different types of electromechanically assisted hand−/arm training after stroke.

We propose that these analyses can be regarded as a supplement to the previous systematic reviews on the topic [3, 4].

The Cochrane review [3] included 44 studies, concluding that there was high quality evidence of small but beneficial effect of robotic arm training on ADL, arm function and strength. High GRADE quality of evidence means that the reviewers are confident that further research will not change the evidence of effect. However, these conclusions relate to the overall effect of robotic arm training, and arise from studies that have used a variety of different robotic devices. Our new network meta-analyses further enhance knowledge in this field by exploring the relative effectiveness of different types of robotic arm training devices. We found evidence that no one category of robotic device was any better or worse than any other device-category. Therefore our results indicate that the type of device (exoskeleton, end-effector, proximal, distal, unilateral etc.) may not be important to patient outcomes.

These findings suggest that there is currently no clear evidence to support the selection of specific types of robotic device as tools to promote hand-arm recovery.

That leaves the clinical practitioner with a dilemma. Currently it is not clear how decisions about the types of robotic device should be made. Our analyses suggests, that the type of electromechanical-assistive device does not matter (which seems to disagree with arguments made by industry, which suggest that different types of device have different actions and will therefore result in different patient outcomes). However, our analyses did not explore whether results varied in sub-groups of patients with different characteristics.

We did not find differences in effects when different tests were used to assess arm function, or when participants had different arm severity or were different times post stroke. This suggests that our main results might be relatively robust and our confidence in these findings is not reduced byknown confounding variables such as duration and severity of stroke. As highlighted earlier, conclusions that can be drawn from previous systematic reviews of arm rehabilitation after stroke have been limited by having a much narrower focus. Our network meta-analyses have sought to address the limitations of previous reviews. The advantages, and novelty, of the work presented here lies in the combination of the results of of randomized controlled trials which have explored various methods of robotic-arm training in one statistical multiple treatment comparison.

### Strengths and limitations

Our pre-planned, rigorous, methods are a key strength of this study. We used a systematic and comprehensive search strategy, and searched a large number of databases for published and unpublished studies. Nevertheless, a publication bias due to non-publication of negative results cannot be ruled out.

Inconsistencies in the description of complex interventions by trial authors can be common [21], and can create challenges to the synthesis and interpretation of evidence. However, the authors pre-planned intervention categorization and statistical comparisons in order to avoid the introduction of bias and post-hoc decisions.

In addition, the heterogeneity of the studies was taken into account by calculating so-called prediction intervals (also called predictive intervals) in addition to confidence intervals. The representation of such predictive intervals is increasingly required for the calculation of effects in meta-analyses, especially in recent times [22].

There are many different important features associated with an electromechanical robotic device. Within our review we categorisedthe electromechanical devices into pre-defined catetories according to whether the device was an end-effector or an exoskeleton (including gloves); and whether the focus was unilateral or bilateral, and on fingers, hand or arm. However there are many other features which will be important to researchers and clinicians with an interest in robotic devices, such as the type of actuation and/or control mechanism. A key limitation of our review is that we have not explored the impact of these features, meaning that our findings are limited to our predefined categories of robotic-approaches, and are unable to answer questions about additional important features of robotic devices.

One may argue that the contents of the control group differed as much as those of the experimental group. However, the authors tried to categorize the therapy description as well as possible on the basis of the information provided by the included studies. The description of the content of the control group was overall rather poorly described, which does limit our confidence in our indirect comparisons.

The authors used arm function as an aspect of severity after stroke and used it in statistical analyses. However, other variables, such as stroke localization, prognostic criteria or handiness, were not included in the evaluation, and these may have influenced the result. However, it is unclear in which direction the result would be biased by this approach.

Mean values of outcomes from the individual studies were used within our study. More exact estimations of treatment effects would be obtained from individual patient data; however, this was beyond the aim of the present study.

We created network diagrams which clearly illustrate the direct comparisons which have been carried out in this field. However, there were an absence of closed loops in the network geometry, which leads to the argument that our analysis is not, in the strictest sense, a NMA or a multiple treatment comparison (MTC), but rather belongs to the NMA genus of Adjusted Indirect Treatment Comparison (ITC) [23].

In our systematic review we did not assess measures of muscle tone of the upper limb. Previous reviews have suggested that this might be negatively influenced by robotic-assisted arm-training [4]. This should be therefore be further investigated.

One limitation of our work is that we have not included other endpoints that are significant for the patient, such as quality of life and participation. In this paper, we concentrated on clinically important endpoints such as everyday activities, hand/arm function and hand/arm strength, which are also important for the patient’s recovery from stroke. However, further studies should focus in particular on other outcomes such as health-related quality of life and social participation.

## Conclusions

The outcomes of robotic-assisted arm training were comparable with conventional therapy.

Indirect comparisons suggest that no one type of robotic device is any better or worse than any other device, and there remains no clear evidence to support the selection of specific types of robotic device to promote hand-arm recovery.

It is important that future studies consider and report key patient characteristics, such as stroke severity and level of upper limb impairment, and important parameters in hand−/arm rehabilitation interventions, such as repetitions, therapy intensity/ -frequency and-increments. Future meta-analyses should incorporate individual patient data in order to support the exploration of the effects of different types of hand−/arm rehabilitation on different populations of patients with stroke.

## Supplementary information

**Additional file 1.** Search strategy for MEDLINE.

**Additional file 2.** Characteristics of included studies.

**Additional file 3.** Risk of bias of included studies.

**Additional file 4.** a-c: Forest plots of indirect comparisons.

**Additional file 5.** Tables of the surface under the cumulative ranking curve (SUCRA) for all outcomes.

**Additional file 6.** Table with description of all adverse events or drop outs occured.

**Additional file 7.** Forest plot of subgroup of studies using the Fugl-Meyer Armtest.

**Additional file 8.** Forest plot of subgroups of studies with three different severities of arm paresis.

**Additional file 9.** Forest plot of subgroups of studies with patients in the first 3 months or later after stroke.

## Data Availability

The datasets supporting the conclusions of this article are included within the article and its additional files.

## Abbreviations

UDFHT: Unilateral distally emphasized finger/hand training
EPAHT: End effector-assisted proximal emphasised unilateral arm/hand training
UPAHT: Unilaterally proximal emphasized arm/hand training
EXAHT: Exoskeleton assisted unilateral arm/hand training
DGFHT: Unilateral distal glove -based finger/hand training
EBAHT: End effector assisted distal and bilateral arm/hand training
95% CI: 95% confidence interval
ADL: Activities of daily living
MD: Mean difference
NMA: Network meta-analysis
RD: Risk difference
SMD: Standardised mean difference
SUCRA: Surface under the cumulative ranking curve
UE-FM: Upper extremity Fugl-Meyer assessment
