# Supplementary material for: Systematic review with network meta-analysis of randomized controlled trials of robotic-assisted arm training for improving activities of daily living and upper limb function after stroke
Source: J Neuroeng Rehabil. 2020 Jun 30;17:83. doi: 10.1186/s12984-020-00715-0 (PMC7325016; doi:10.1186/s12984-020-00715-0)
Supplement: Supplementary file 1 — Additional file 1. Search strategy for MEDLINE. [file 12984_2020_715_MOESM1_ESM.pdf]

Additional file 1: search strategy

This search strategy was for MEDLINE via Ovid and was adapted for the other databases.

1. cerebrovascular disorders/ or exp basal ganglia cerebrovascular disease/ or exp brain ischemia/ or exp carotid artery diseases/ or exp cerebral small vessel diseases/ or exp intracranial arterial diseases/ or exp "intracranial embolism and thrombosis"/ or exp intracranial hemorrhages/ or stroke/ or exp brain infarction/ or stroke, lacunar/ or vasospasm, intracranial/ or vertebral artery dissection/ or brain injuries/ or brain injury, chronic/
2. (stroke$ or poststroke or apoplex$ or cerebral vasc$ or brain vasc$ or cerebrovasc$ or cva$ or SAH).tw.
3. ((brain or cerebr$ or cerebell$ or vertebrobasil$ or hemispher$ or intracran$ or intracerebral or infratentorial or supratentorial or middle cerebral artery or MCA$ or anterior circulation or posterior circulation or basilar artery or vertebral artery or space-occupying) adj5 (isch?emi$ or infarct$ or thrombo$ or emboli$ or occlus$ or hypoxi$)).tw.
4. ((brain$ or cerebr$ or cerebell$ or intracerebral or intracran$ or parenchymal or intraparenchymal or intraventricular or infratentorial or supratentorial or basal gangli$ or putaminal or putamen or posterior fossa or hemispher$ or subarachnoid) adj5 (h?emorrhag$ or h?ematoma$ or bleed$)).tw.
5. hemiplegia/ or exp paresis/
6. (hemipleg$ or hemipar$ or paresis or paretic or brain injur$).tw.
7. or/1-6
8. exp upper extremity/
9. (upper limb$ or upper extremit$ or arm or arms or shoulder or shoulders or hand or hands or axilla$ or elbow$ or forearm$ or finger$ or wrist$).tw.
10. 8 or 9
11. robotics/ or automation/ or orthotic devices/
12. "equipment and supplies"/ or self-help devices/
13. physical therapy modalities/ or occupational therapy/
14. therapy, computer-assisted/ or man-machine systems/
15. exercise movement techniques/ or exercise/ or exercise therapy/ or muscle stretching techniques/ or motion therapy, continuous passive/
16. (robot$ or orthos$ or orthotic or automat$ or computer aided or computer assisted or device$).tw.
17. (electromechanical or electro-mechanical or mechanical or mechanised or mechanized or driven).tw.
18. ((continuous passive or cpm) adj3 therap$).tw.
19. (MIT-Manus or ARM guide or Bi-Manu-Track or ARMtrainer or MIME or Mirror-Image Motion Enabler or InMOTION or REHAROB or NeReBot or "GENTLE/S" or ARMin).tw.
20. (assist$ adj5 (train$ or aid$ or rehabilitat$ or re-educat$)).tw.
21. or/11-20
22. Randomized Controlled Trials as Topic/
23. random allocation/
24. Controlled Clinical Trials as Topic/
25. control groups/
26. clinical trials as topic/ or clinical trials, phase i as topic/ or clinical trials, phase ii as topic/ or clinical trials, phase iii as topic/ or clinical trials, phase iv as topic/
27. double-blind method/
28. single-blind method/
29. Placebos/
30. placebo effect/
31. cross-over studies/
32. randomized controlled trial.pt.
33. controlled clinical trial.pt.
34. (clinical trial or clinical trial phase i or clinical trial phase ii or clinical trial phase iii or clinical trial phase iv).pt.
35. (random$ or RCT or RCTs).tw.

36. (controlled adj5 (trial$ or stud$)).tw.
37. (clinical$ adj5 trial$).tw.
38. ((control or treatment or experiment$ or intervention) adj5 (group$ or subject$ or patient$)).tw.
39. (quasi-random$ or quasi random$ or pseudo-random$ or pseudo random$).tw.
40. ((control or experiment$ or conservative) adj5 (treatment or therapy or procedure or manage$)).tw.
41. ((singl$ or doubl$ or tripl$ or trebl$) adj5 (blind$ or mask$)).tw.
42. (cross-over or cross over or crossover).tw.
43. (placebo$ or sham).tw.
44. trial.ti.
45. (assign$ or allocat$).tw.
46. controls.tw.
47. or/22-46
48. 7 and 10 and 21 and 47
49. exp animals/ not humans.sh.
50. 48 not 49
