# Supplementary material for: Systematic review with network meta-analysis of randomized controlled trials of robotic-assisted arm training for improving activities of daily living and upper limb function after stroke
Source: J Neuroeng Rehabil. 2020 Jun 30;17:83. doi: 10.1186/s12984-020-00715-0 (PMC7325016; doi:10.1186/s12984-020-00715-0)
Supplement: Supplementary file 2 — Additional file 2. Characteristics of included studies. [file 12984_2020_715_MOESM2_ESM.pdf]

Additional file 2: characteristics of studies

Study ID	Intervention	device	duration of device intervention	frequency	n	duration of illness (device group)	duration of control treatment	frequency of control treatment	n	duration of illness control	Assessment Function
Abdullah 2011	UPAHT	industrial robot	9 weeks	0,75h three times per week	9	1	9 weeks	0,75h three times per week	11	1	CAHAI-7
Ang 2014	DGFHT	Haptic knob	6 weeks	1,5h three times per week	14	12	6 weeks	1,5h three times per week	7	15	UE-FM
Brokaw 2014	EXAHT	ARMin III, HandSOME	12 weeks	12h per month	7	36	12 weeks	12h per month	5	36	UE-FM
Burgar 2011	UPAHT	MIME	3 weeks	1,5h five times per week	36	1	3 weeks	0,67h five times per week	18	1	UE-FM
Bustamante 2016	UDFHT	Robot Gym (TheraDrive)	7 weeks	24 sessions a 2h	10	NA	7 weeks	24 sessions a 2h	10	NA	UE-FM
Conroy 2011	UPAHT	InMotion 2.0	6 weeks	0,5h three times per week	38	48	6 weeks	0,5h three times per week	19	48	UE-FM
Daly 2005	UPAHT	InMotion	12 weeks	5h five times per week	7	NA	12 weeks	5h five times per week	6	NA	UE-FM
Fazekas 2007	EPAHT	REHABROB	5 weeks	0,5h five times per week	15	23	5 weeks	0,5h five times per week	15	10	UE-FM
Grigoras 2016	DGFHT	Hybrid FES-Exoskelett	2 weeks	0,5h sechsmal per week	13	4	2 weeks	0,5h five times per week	12	4	UE-FM
Hesse 2005	EBAHT	Bi-Manu Track	6 weeks	0,5h five times per week	22	1	6 weeks	0,5h five times per week	22	1	UE-FM
Hesse 2014	EBAHT	Bi-Manu Track	4 weeks	0,5h five times per week	25	1	4 weeks	0,5h five times per week	25	1	UE-FM
Hollenstein 2011	EXAHT	Armeo	2 weeks	0,5h five times per week	7	1	2 weeks	0,5h five times per week	6	1	UE-FM
Housman 2009	EXAHT	T-WREX	8,5 weeks	1h three times per week	17	12	8,5 weeks	1h three times per week	17	12	UE-FM
Hsieh 2011	EBAHT	Bi-Manu Track	4 weeks	1,5h five times per week	13	17	4 weeks	1,5h five times per week	6	28	UE-FM
Hsieh 2014	EBAHT	Bi-Manu Track	4 weeks	1,5h five times per week	32	22	4 weeks	1,5h five times per week	16	28	UE-FM
Hwang 2012	UDFHT	Amadeo	4 weeks	0,75h five times per week	9	7	4 weeks	0,75h five times per week	6	5	UE-FM
KlamrothMarganska 2014	EXAHT	ARMin	8 weeks	0,75h three times per week	39	52	8 weeks	0,75h three times per week	38	40	UE-FM
Kutner 2010	UDFHT	Handmentor	3 weeks	4h five times per week	10	9	3 weeks	4h five times per week	11	6	SIS hand function
Lee 2016	UPAHT	NEURO X System	2 weeks	1h five times per week	22	1	2 weeks	1h five times per week	22	1	Manual function test

Liao 2011	EBAHT	Bi-Manu Track	4 weeks	1,5h five times per week	10	23		4 weeks	1,5h five times per week	10	22	UE-FM
Lo 2010	UPAHT	MIT Manus	12 weeks	36 sessions	49	4		12 weeks	36 sessions	78	5	UE-FM
Lum 2006	UPAHT	MIME	4 weeks	1h four times per week	24	30		4 weeks	1h four times per week	6	29	UE-FM
Masiero 2007	EPAHT	NEREBOT	5 weeks	0,4h zehnmal per week	17	1		5 weeks	0,4h ten times per week	18	1	UE-FM
Masiero 2011	EPAHT	NEREBOT	5 weeks	1,3h five times per week	11	1		5 weeks	1,3h five times per week	10	1	UE-FM
Mayr 2008	EXAHT	ARMOR	6 weeks	1,5h five times per week	4	NA		6 weeks	1h five times per week	4	NA	CMSA
McCabe 2015	UPAHT	InMotion 2.0	5 weeks	5h five times per week	12	NA		5 weeks	5h five times per week	23	NA	UE-FM
Orihuela-Espina 2016	UDFHT	Amadeo	9 weeks	1h five times per week	9	NA		9 weeks	1h five times per week	8	NA	UE-FM
Rabadi 2008	UPAHT	MIT-Manus	Unklar	3,67h five times per week	20	1		Unklar	3,67h five times per week	10	1	UE-FM
Sale 2014	UPAHT	MIT-Manus/InMotion 2.0	6 weeks	3,75h five times per week	26	NA		6 weeks	3h five times per week	27	NA	UE-FM
Stein 2017	UDFHT	Amadeo	8 weeks	five times per week	14	3		8 weeks	five times per week	14	3	UE-FM
Susanto 2015	DGFHT	Hand-exoskelett (self made)	5 weeks	1h four times per week	9	16		5 weeks	1h four times per week	10	16	UE-FM
Takahashi 2016	UPAHT	Reogo	6 weeks	0,67h seven times per week	30	2		6 weeks	0,67h seven times per week	26	2	UE-FM
Timmermans 2014	EPAHT	HapticMaster	8 weeks	1h four times per week	11	36		8 weeks	1h four times per week	11	48	UE-FM
Tomic 2017	UPAHT	Armassist	3 weeks	0,5h five times per week	13	1		3 weeks	0,5h five times per week	13	1	UE-FM
Vanoglio 2017	DGFHT	Gloreha	4 weeks	0,75h five times per week	14	0,5		4 weeks	0,75h five times per week	13	0,5	Quick-DASH
Villafane 2017	DGFHT	Gloreha	3 weeks	0,5h five times per week	16	6		3 weeks	0,5h five times per week	16	6	Quick-DASH
Volpe 2000	UPAHT	MIT-Manus	5 weeks	1h five times per week	30	1		5 weeks	1h five times per week	26	1	UE-FM
Volpe 2008	UPAHT	In Motion 2.0	6 weeks	1h three times per week	11	35		6 weeks	1h three times per week	10	40	UE-FM
Wolf 2015	UDFHT	Hand Mentor per	8 weeks	3h five times per week	51	4		8 weeks	3h five times per week	48	4	UE-FM
Wu 2012	EBAHT	Bi-Manu Track	4 weeks	1,5h five times per week	14	18		4 weeks	1,5h five times per week	28	18	UE-FM
Yoo 2013	UPAHT	Reogo	6 weeks	1,5h three times per week	11	46		6 weeks	1,5h three times per week	11	42	WMFT

Cho 2019	UPAHT	RT-AAN	6 weeks	40 minutes three times per week	21	2,5	6 weeks	40 minutes three times per week	21	2,75	UE_FM
Qian 2017	EPAHT	NMES ROBOT	4 weeks	1h five times per week	14	4	4 weeks	1h five times per week	10	4	UE_FM
Hung 2019	UPAHT	Inmotion	4 weeks	1,5h five times per week	10	26	4 weeks	1,5h five times per week	10	25	UE_FM
Hung 2019	EBAHT	Bi-Manu Track	4 weeks	1,5h five times per week	10	20,5	4 weeks	1,5h five times per week	10	25	UE_FM
Daun 2018	EXAHT	Armeo Spring	2 weeks	0,5h five times per week	17	2,25	2 weeks	0,5h five times per week	17	2,5	UE_FM
Kim 2019	EPAHT	RA- Shoulder therapy	4 weeks	0,5h five times per week	19	3,25	4 weeks	0,5h five times per week	19	3,25	K_SDQ
Lee 2018	EPAHT	REJOYCE Rob	8 weeks	0,5h five times per week	15	6	8 weeks	0,5h five times per week	15	6	UE_FM
RATULS 2019	UPAHT	MIT-Manus	12 weeks	45 minutes three times per week	257	8,25	12 weeks	45 minutes three times per week	###	9	UE_FM
Conroy2019	UPAHT	Inmotion	12 weeks	45 minutes three times per week	22	80	12 weeks	45 minutes three times per week	23	140	UE_FM
