# Supplementary material for: Systematic review with network meta-analysis of randomized controlled trials of robotic-assisted arm training for improving activities of daily living and upper limb function after stroke
Source: J Neuroeng Rehabil. 2020 Jun 30;17:83. doi: 10.1186/s12984-020-00715-0 (PMC7325016; doi:10.1186/s12984-020-00715-0)
Supplement: Supplementary file 3 — Additional file 3. Risk of bias of included studies. [file 12984_2020_715_MOESM3_ESM.pdf]

Additional file 3: Risk of bias of included studies

**Study ID**	**Risk of Bias: Randomisation**	**Risk of Bias: Concealment of Allocation**	**Risk of Bias: Blinding of Assessors**
Abdullah 2011	low	unclear	low
Ang 2014	low	unclear	low
Brokaw 2014	low	unclear	low
Burgar 2011	low	unclear	low
Bustamante 2016	low	unclear	high
Conroy 2011	low	unclear	low
Daly 2005	unclear	unclear	low
Fazekas 2007	unclear	unclear	low
Grigoras 2016	unclear	unclear	high
Hesse 2005	low	low	low
Hesse 2005	low	low	low
Hesse 2014	low	unclear	low
Hesse 2014	low	unclear	low
Hollenstein 2011	low	unclear	unclear
Housman 2009	low	low	low
Hsieh 2011	low	low	low
Hsieh 2014	low	low	low
Hwang 2012	low	unclear	low
KlamrothMarganska 2014	low	low	low
Kutner 2010	low	unclear	low
Lee 2016	low	low	high
Liao 2011	low	low	low
Lo 2010	low	unclear	low
Lum 2006	unclear	unclear	low
Masiero 2007	unclear	unclear	low
Masiero 2007	unclear	unclear	low
Masiero 2011	low	unclear	low
Masiero 2011	low	unclear	low
Mayr 2008	low	high	high
Mayr 2008	low	high	high
McCabe 2015	unclear	unclear	low
Orihuela-Espina 2016	low	unclear	high
Rabadi 2008	low	low	low
Sale 2014	unclear	unclear	low
Stein 2017	unclear	unclear	high
Susanto 2015	low	unclear	low
Takahashi 2016	low	low	low
Timmermans 2014	low	low	low
Tomic 2017	low	unclear	low
Vanoglio 2017	low	low	low
Villafane 2017	unclear	unclear	low
Volpe 2000	unclear	unclear	high
Volpe 2008	unclear	unclear	high
Wolf 2015	low	unclear	low
Wu 2012	unclear	low	low
Yoo 2013	unclear	unclear	low

Cho 2019	low	low	high
Qian 2017	unclear	unclear	high
Hung 2019	low	unclear	low
Daun 2018	unclear	unclear	high
Kim 2019	unclear	unclear	high
Lee 2018	unclear	unclear	low
RATULS 2019	low	low	low
Conroy2019	unclear	unclear	low
