# Supplementary figures and images for: Systematic review with network meta-analysis of randomized controlled trials of robotic-assisted arm training for improving activities of daily living and upper limb function after stroke
Source: J Neuroeng Rehabil. 2020 Jun 30;17:83. doi: 10.1186/s12984-020-00715-0 (PMC7325016; doi:10.1186/s12984-020-00715-0)

## Treatment Effect

## Mean with 95%CI and 95%PrI

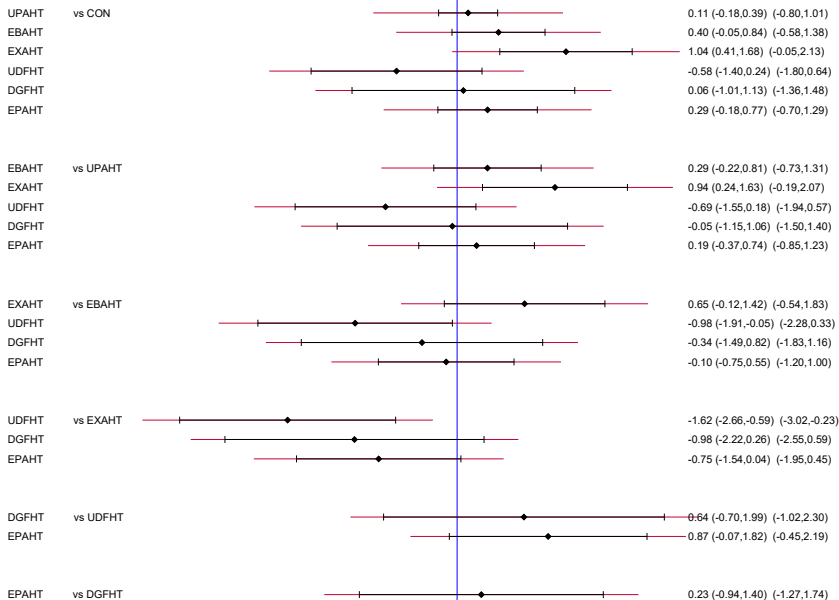

-3

-1.7

0

1

2.3

Supplement: Supplementary file 4 — Additional file 4. a-c: Forest plots of indirect comparisons. [file 12984_2020_715_MOESM4_ESM.zip › Additional file 4a ADL_plot_Indirect comparisons.pdf]

# Treatment Effect

# Mean with 95%CI and 95%PrI

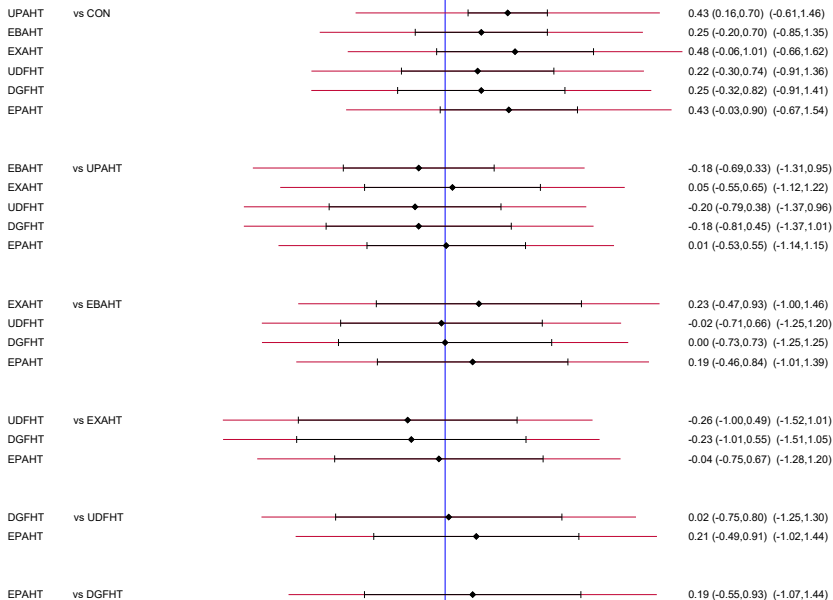

Supplement: Supplementary file 4 — Additional file 4. a-c: Forest plots of indirect comparisons. [file 12984_2020_715_MOESM4_ESM.zip › Additional file 4b Function_plot_Indirect comparisons.pdf]

## Treatment Effect

## Mean with 95%CI and 95%Pr

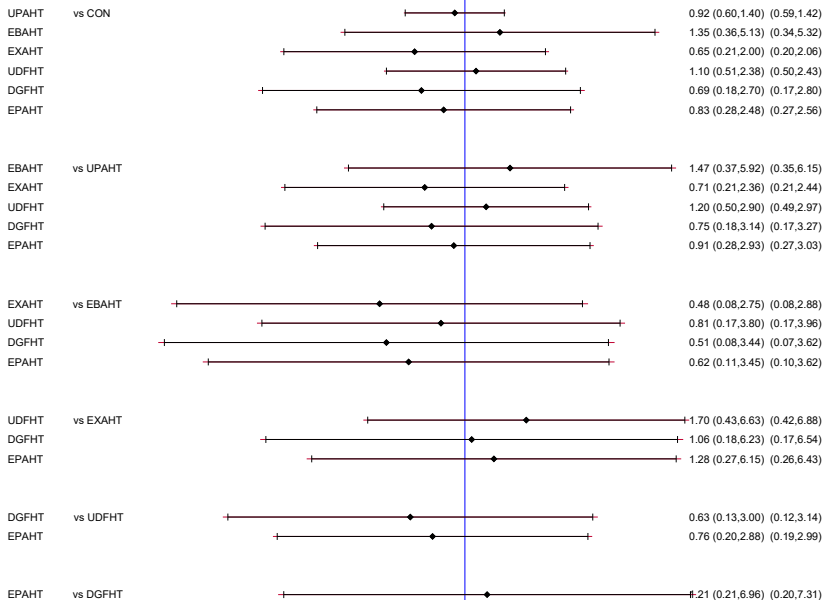

Supplement: Supplementary file 4 — Additional file 4. a-c: Forest plots of indirect comparisons. [file 12984_2020_715_MOESM4_ESM.zip › Additional file 4c AE_plot_Indirect comparisons.pdf]

## Reference treatment: CON

Treatment Effect

Mean with 95%CI and 95%PrI

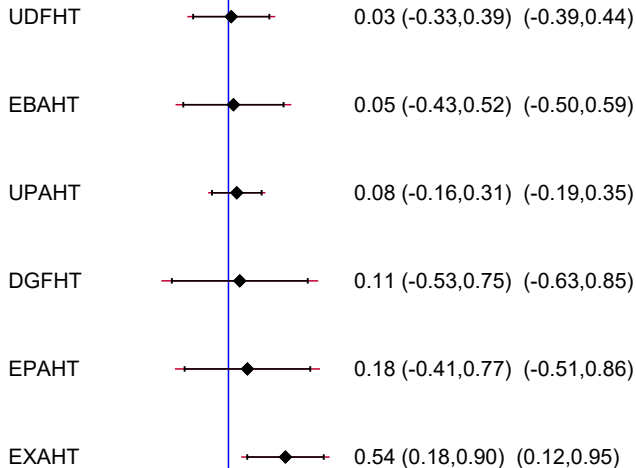

-0.6 -0.20 .6 1

Supplement: Supplementary file 8 — Additional file 8. Forest plot of subgroups of studies with three different severities of arm paresis. [file 12984_2020_715_MOESM8_ESM.zip › AF8b subgroup analysis UE-FM between 20 and 40.pdf]

# Reference treatment: CON

Treatment Effect

Mean with 95%CI and 95%PrI

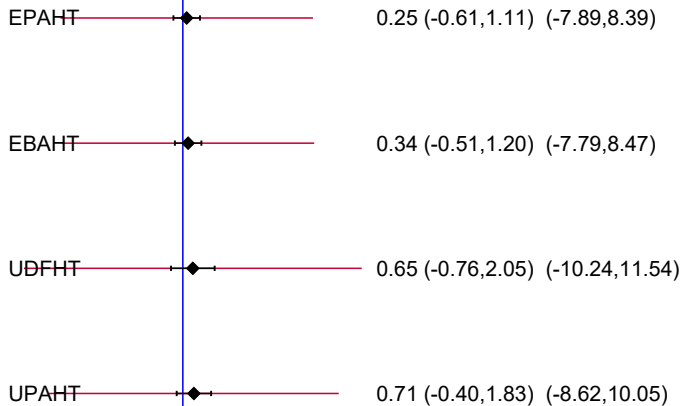

-10 -4.8 0 6.1 12

Supplement: Supplementary file 8 — Additional file 8. Forest plot of subgroups of studies with three different severities of arm paresis. [file 12984_2020_715_MOESM8_ESM.zip › AF8c subgroup analysis UE-FM more than 40.pdf]
