# Supplementary material for: Systematic review with network meta-analysis of randomized controlled trials of robotic-assisted arm training for improving activities of daily living and upper limb function after stroke
Source: J Neuroeng Rehabil. 2020 Jun 30;17:83. doi: 10.1186/s12984-020-00715-0 (PMC7325016; doi:10.1186/s12984-020-00715-0)
Supplement: Supplementary file 5 — Additional file 5. Tables of the surface under the cumulative ranking curve (SUCRA) for all outcomes. [file 12984_2020_715_MOESM5_ESM.pdf]

Additional file 5: tables of the surface under the cumulative ranking curve (SUCRA) for all outc

Primary Outcome: activities of daily living

Intervention	SUCRA (P-Score)
EXAHT	97,4
EBAHT	70,3
EPAHT	59,7
UPAHT	43,9
DGFHT	43,4
CON	29,3
UDFHT	5,9

Primary Outcome: hand-arm function

Intervention	SUCRA (P-Score)
EXAHT	70,4
UPAHT	68,9
EPAHT	67,9
EBAHT	45,5
DGFHT	43,7
UDFHT	43,5
CON	10,2

Secondary outcome: adverse events

Intervention	SUCRA (P-Score)
EXAHT	70,5
DGFHT	63,3
EPAHT	57,9
UPAHT	51,4
CON	41,8
UDFHT	35,8
EBAHT	29,3

:omes
