# Supplementary material for: Systematic review with network meta-analysis of randomized controlled trials of robotic-assisted arm training for improving activities of daily living and upper limb function after stroke
Source: J Neuroeng Rehabil. 2020 Jun 30;17:83. doi: 10.1186/s12984-020-00715-0 (PMC7325016; doi:10.1186/s12984-020-00715-0)
Supplement: Supplementary file 6 — Additional file 6. Table with description of all adverse events or drop outs occured. [file 12984_2020_715_MOESM6_ESM.docx]

**Additional file 6:**

**Adverse events in all included studies (RA: robotic assistant arm-training)**

| **Study-ID** | **Intervention** | **study** | **events** | **total** |
| --- | --- | --- | --- | --- |
| Abdullah 2011 | RA | 1 | 1 | 9 |
| Abdullah 2011 | Control | 1 | 0 | 11 |
| Ang 2014 | RA | 2 | 1 | 15 |
| Ang 2014 | Control | 2 | 0 | 7 |
| Brokaw 2014 | RA | 3 | 0 | 7 |
| Brokaw 2014 | Control | 3 | 2 | 5 |
| Burgar 2011 | RA | 4 | 0 | 36 |
| Burgar 2011 | Control | 4 | 0 | 18 |
| Bustamante 2016 | RA | 5 | 3 | 13 |
| Bustamante 2016 | Control | 5 | 4 | 14 |
| Conroy 2011 | RA | 6 | 5 | 41 |
| Conroy 2011 | Control | 6 | 2 | 21 |
| Daly 2005 | RA | 7 | 1 | 7 |
| Daly 2005 | Control | 7 | 0 | 6 |
| Fazekas 2007 | RA | 8 | 0 | 15 |
| Fazekas 2007 | Control | 8 | 0 | 15 |
| Grigoras 2016 | RA | 9 | 0 | 13 |
| Grigoras 2016 | Control | 9 | 0 | 12 |
| Hesse 2005 | RA | 10 | 1 | 22 |
| Hesse 2005 | Control | 10 | 0 | 22 |
| Hesse 2014 | RA | 11 | 1 | 25 |
| Hesse 2014 | Control | 11 | 0 | 25 |
| Hollenstein 2011 | RA | 12 | 0 | 7 |
| Hollenstein 2011 | Control | 12 | 0 | 6 |
| Housman 2009 | RA | 13 | 2 | 17 |
| Housman 2009 | Control | 13 | 1 | 17 |
| Hsieh 2011 | RA | 14 | 0 | 13 |
| Hsieh 2011 | Control | 14 | 0 | 6 |
| Hsieh 2014 | RA | 15 | 0 | 32 |
| Hsieh 2014 | Control | 15 | 0 | 16 |
| Hu 2015 | RA | 16 | 0 | 26 |
| Hwang 2012 | RA | 17 | 0 | 9 |
| Hwang 2012 | Control | 17 | 2 | 8 |
| Kahn 2006 | RA | 18 | 0 | 10 |
| Kahn 2006 | Control | 18 | 0 | 9 |
| KlamrothMarganska 2014 | RA | 19 | 1 | 39 |
| KlamrothMarganska 2014 | Control | 19 | 3 | 38 |
| Kutner 2010 | RA | 20 | 3 | 10 |
| Kutner 2010 | Control | 20 | 1 | 11 |
| Lee 2016 | RA | 21 | 7 | 29 |
| Lee 2016 | Control | 21 | 7 | 29 |
| Liao 2011 | RA | 22 | 0 | 10 |
| Liao 2011 | Control | 22 | 0 | 10 |
| Lo 2010 | RA | 23 | 5 | 49 |
| Lo 2010 | Control | 23 | 11 | 78 |
| Lum 2002 | RA | 24 | 2 | 15 |
| Lum 2002 | Control | 24 | 1 | 15 |
| Lum 2006 | RA | 25 | 0 | 24 |
| Lum 2006 | Control | 25 | 0 | 6 |
| Masiero 2007 | RA | 26 | 2 | 17 |
| Masiero 2007 | Control | 26 | 3 | 18 |
| Masiero 2011 | RA | 27 | 0 | 11 |
| Masiero 2011 | Control | 27 | 0 | 10 |
| Mayr 2008 | RA | 28 | 0 | 4 |
| Mayr 2008 | Control | 28 | 0 | 4 |
| McCabe 2015 | Control | 29 | 0 | 23 |
| McCabe 2015 | RA | 29 | 0 | 12 |
| OrihuelaEspina 2016 | RA | 30 | 0 | 9 |
| OrihuelaEspina 2016 | Control | 30 | 0 | 8 |
| Rabadi 2008 | Control | 31 | 0 | 20 |
| Rabadi 2008 | RA | 31 | 0 | 10 |
| Sale 2014 | RA | 32 | 0 | 26 |
| Sale 2014 | Control | 32 | 0 | 27 |
| Stein 2017 | RA | 33 | 2 | 16 |
| Stein 2017 | Control | 33 | 1 | 15 |
| Susanto 2015 | RA | 34 | 0 | 9 |
| Susanto 2015 | Control | 34 | 1 | 10 |
| Takahashi 2016 | RA | 35 | 0 | 30 |
| Takahashi 2016 | Control | 35 | 4 | 30 |
| Taveggia 2016 | RA | 36 | 0 | 27 |
| Taveggia 2016 | Control | 36 | 0 | 27 |
| Timmermanns 2014 | RA | 37 | 0 | 11 |
| Timmermanns 2014 | Control | 37 | 0 | 11 |
| Tomic 2017 | RA | 38 | 0 | 13 |
| Tomic 2017 | Control | 38 | 0 | 13 |
| Tropea 2013 | RA | 39 | 0 | 18 |
| Vanoglio 2017 | RA | 40 | 1 | 15 |
| Vanoglio 2017 | Control | 40 | 2 | 15 |
| Villafane 2017 | RA | 41 | 0 | 16 |
| Villafane 2017 | Control | 41 | 0 | 16 |
| Volpe 2000 | RA | 42 | 0 | 30 |
| Volpe 2000 | Control | 42 | 0 | 26 |
| Volpe 2008 | RA | 43 | 0 | 11 |
| Volpe 2008 | Control | 43 | 0 | 10 |
| Wolf 2015 | RA | 44 | 4 | 51 |
| Wolf 2015 | Control | 44 | 3 | 48 |
| Wu 2012 | RA | 45 | 0 | 14 |
| Wu 2012 | Control | 45 | 0 | 28 |
| Yoo 2013 | RA | 46 | 0 | 11 |
| Yoo 2013 | Control | 46 | 0 | 11 |
| Cho 2019 | RA | 47 | 2 | 21 |
| Cho 2019 | Control | 47 | 2 | 21 |
| Qian 2017 | RA | 48 | 0 | 14 |
| Qian 2017 | Control | 48 | 0 | 10 |
| Hung 2019 | RA | 49 | 0 | 10 |
| Hung 2019 | RA | 49 | 0 | 10 |
| Hung 2019 | Control | 49 | 0 | 10 |
| Daun 2018 | RA | 50 | 0 | 17 |
| Daun 2018 | Control | 50 | 0 | 17 |
| Iwamoto 2019 | RA | 51 | 0 | 6 |
| Iwamoto 2019 | Control | 51 | 0 | 6 |
| Kim 2019 | RA | 52 | 1 | 19 |
| Kim 2019 | Control | 52 | 1 | 19 |
| Lee 2018 | RA | 53 | 0 | 15 |
| Lee 2018 | Control | 53 | 0 | 15 |
| RATULS 2019 | RA | 54 | 5 | 257 |
| RATULS 2019 | Control | 54 | 17 | 513 |
