# Supplementary material for: Systematic review with network meta-analysis of randomized controlled trials of robotic-assisted arm training for improving activities of daily living and upper limb function after stroke
Source: J Neuroeng Rehabil. 2020 Jun 30;17:83. doi: 10.1186/s12984-020-00715-0 (PMC7325016; doi:10.1186/s12984-020-00715-0)
Supplement: Supplementary file 7 — Additional file 7. Forest plot of subgroup of studies using the Fugl-Meyer Armtest. [file 12984_2020_715_MOESM7_ESM.pdf]

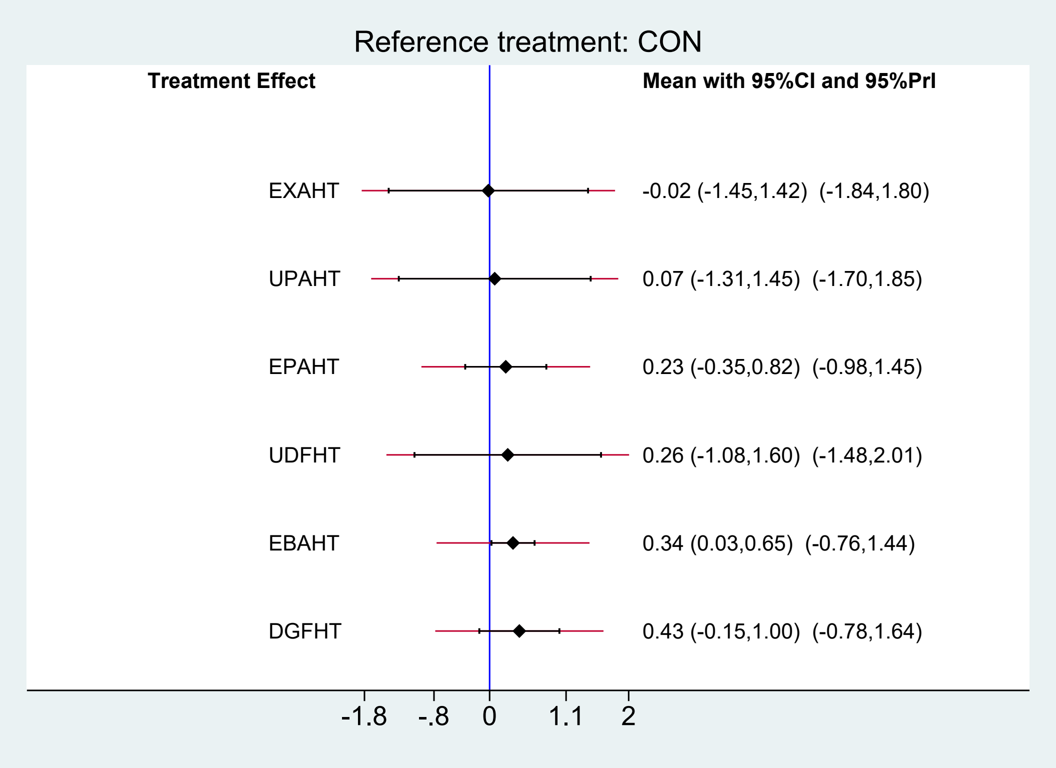
Reference treatment: CON
Treatment Effect
Mean with 95%CI and 95%PrI
EXAHT
-0.02 (-1.45,1.42) (-1.84,1.80)
UPAHT
0.07 (-1.31,1.45) (-1.70,1.85)
EPAHT
0.23 (-0.35,0.82) (-0.98,1.45)
UDFHT
0.26 (-1.08,1.60) (-1.48,2.01)
EBAHT
0.34 (0.03,0.65) (-0.76,1.44)
DGFHT
0.43 (-0.15,1.00) (-0.78,1.64)
-1.8
-.8
0
1.1
2
