# Supplementary material for: Systematic review with network meta-analysis of randomized controlled trials of robotic-assisted arm training for improving activities of daily living and upper limb function after stroke
Source: J Neuroeng Rehabil. 2020 Jun 30;17:83. doi: 10.1186/s12984-020-00715-0 (PMC7325016; doi:10.1186/s12984-020-00715-0)
Supplement: Supplementary file 8 — Additional file 8. Forest plot of subgroups of studies with three different severities of arm paresis. [file 12984_2020_715_MOESM8_ESM.zip › AF8a subgroup analysis UE-FM less than 20.pdf]

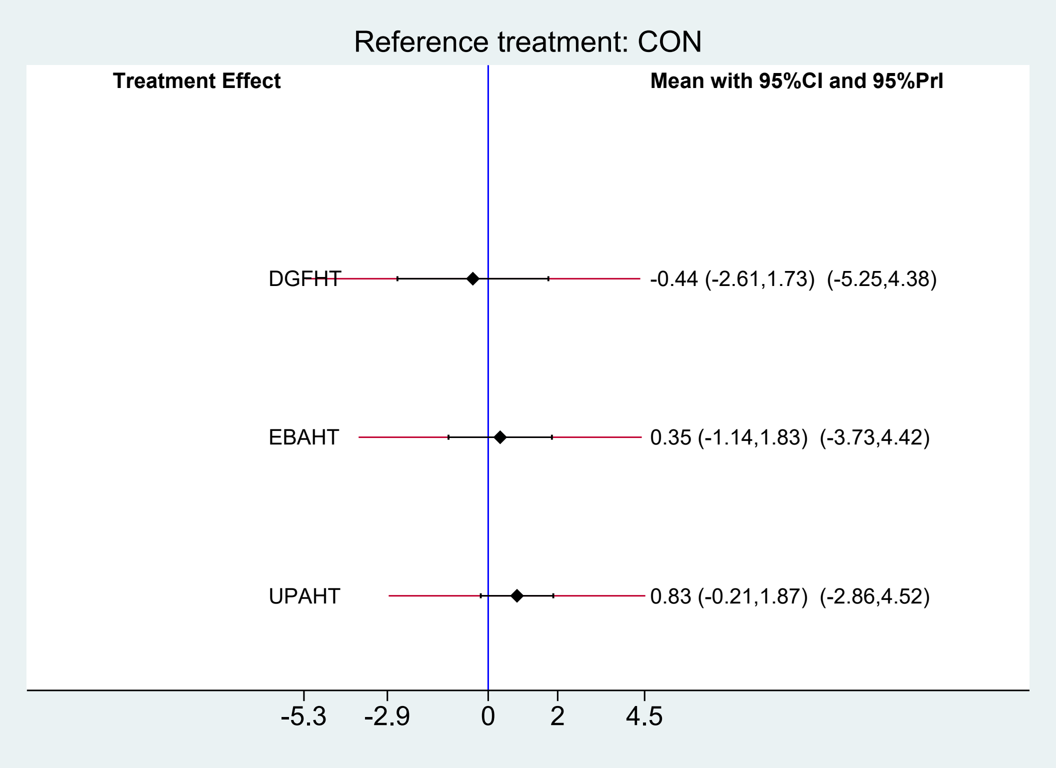

Reference treatment: CON
Treatment Effect
Mean with 95%CI and 95%PrI
DGFHT
-0.44 (-2.61,1.73) (-5.25,4.38)
EBAHT
0.35 (-1.14,1.83) (-3.73,4.42)
UPAHT
0.83 (-0.21,1.87) (-2.86,4.52)
-5.3
-2.9
0
2
4.5
