# Supplementary material for: Systematic review with network meta-analysis of randomized controlled trials of robotic-assisted arm training for improving activities of daily living and upper limb function after stroke
Source: J Neuroeng Rehabil. 2020 Jun 30;17:83. doi: 10.1186/s12984-020-00715-0 (PMC7325016; doi:10.1186/s12984-020-00715-0)
Supplement: Supplementary file 9 — Additional file 9. Forest plot of subgroups of studies with patients in the first 3 months or later after stroke. [file 12984_2020_715_MOESM9_ESM.zip › AF9a subgroup less than 3months post stroke.pdf]

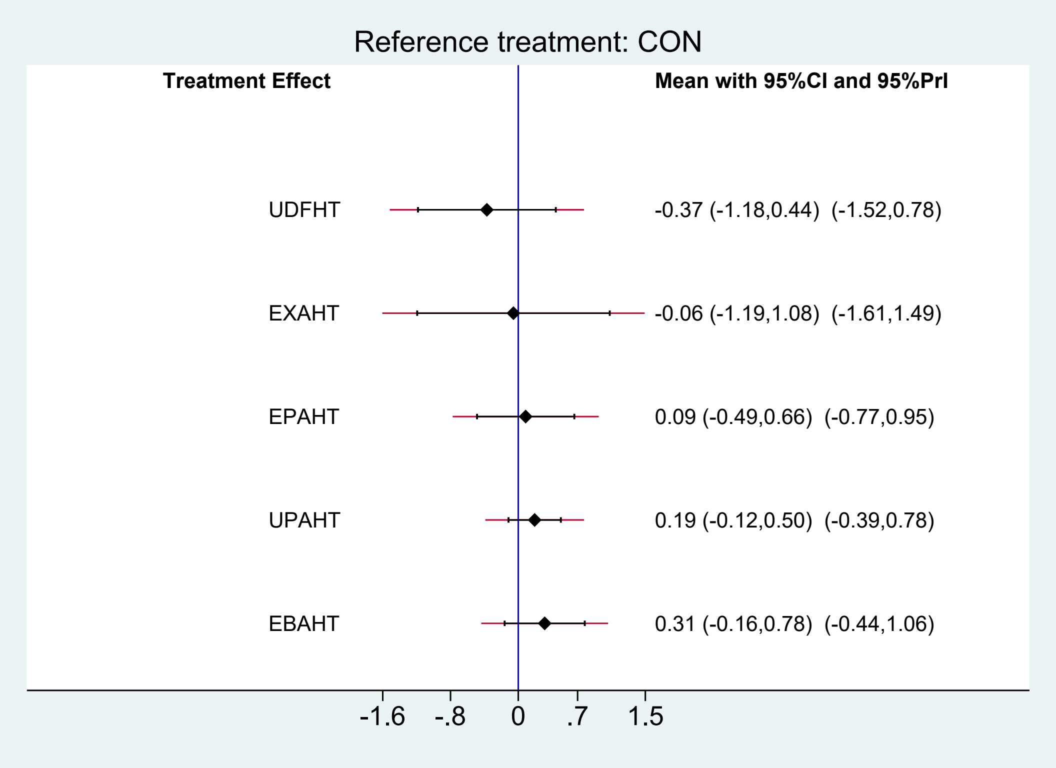

Reference treatment: CON
Treatment Effect
Mean with 95%CI and 95%PrI
UDFHT
-0.37 (-1.18,0.44) (-1.52,0.78)
EXAHT
-0.06 (-1.19,1.08) (-1.61,1.49)
EPAHT
0.09 (-0.49,0.66) (-0.77,0.95)
UPAHT
0.19 (-0.12,0.50) (-0.39,0.78)
EBAHT
0.31 (-0.16,0.78) (-0.44,1.06)
-1.6
-.8
0
.7
1.5
