# Supplementary material for: Systematic review with network meta-analysis of randomized controlled trials of robotic-assisted arm training for improving activities of daily living and upper limb function after stroke
Source: J Neuroeng Rehabil. 2020 Jun 30;17:83. doi: 10.1186/s12984-020-00715-0 (PMC7325016; doi:10.1186/s12984-020-00715-0)
Supplement: Supplementary file 9 — Additional file 9. Forest plot of subgroups of studies with patients in the first 3 months or later after stroke. [file 12984_2020_715_MOESM9_ESM.zip › AF9b subgroup more than 3months post stroke.pdf]

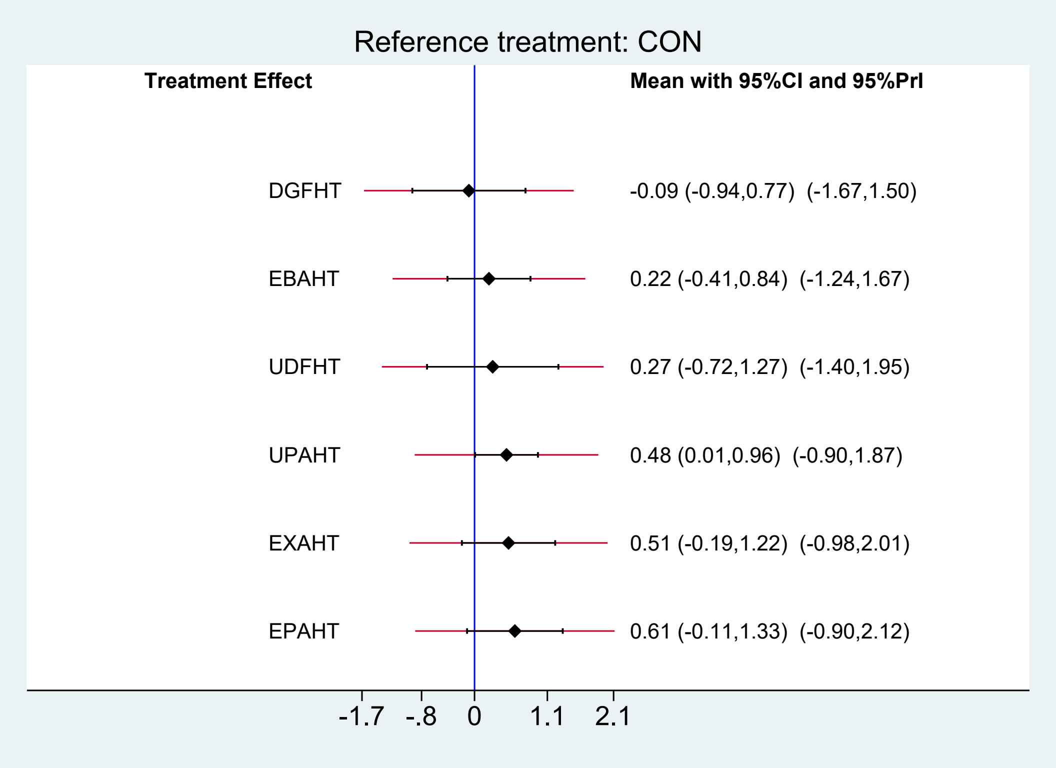
Reference treatment: CON
Treatment Effect
Mean with 95%CI and 95%PrI
DGFHT
-0.09 (-0.94,0.77) (-1.67,1.50)
EBAHT
0.22 (-0.41,0.84) (-1.24,1.67)
UDFHT
0.27 (-0.72,1.27) (-1.40,1.95)
UPAHT
0.48 (0.01,0.96) (-0.90,1.87)
EXAHT
0.51 (-0.19,1.22) (-0.98,2.01)
EPAHT
0.61 (-0.11,1.33) (-0.90,2.12)
-1.7
-.8
0
1.1
2.1
